# CXCL16 Induces the Progression of Pulmonary Fibrosis through Promoting the Phosphorylation of STAT3

**DOI:** 10.1155/2019/2697376

**Published:** 2019-07-10

**Authors:** Sheng Zuo, Zhen Zhu, Yi Liu, Hong Li, Shuang Song, Shaojun Yin

**Affiliations:** Department of Respiration, Shanghai Sixth People's Hospital East Affiliated to Shanghai University of Medicine & Health Sciences, Shanghai, China

## Abstract

**Aim:**

The transmembrane chemokine (C-X-C motif) ligand 16 (CXCL16) plays a vital role in the pathogenesis of organ fibrosis, including the liver and kidney. However, the detailed biological function of CXCL16 is still not fully understood in the progression of pulmonary fibrosis (PF). The aim of present study is to examine the function of CXCL16 in PF.

**Materials and Methods:**

In this study, we constructed the PF model on mouse by using bleomycin and analyzed the effect of the mouse recombinant protein CXCL16 on mouse lung fibroblast L929 (LF) as well. To further examine the connection between CXCL16 and STAT3 in mouse LF cells, the STAT3 inhibitor AG490 was utilized to inhibit the expression of STAT3. Meanwhile, lipopolysaccharide was used to enhance the phosphorylation of STAT3 (p-STAT3) in mouse LF cells.

**Results:**

Our results indicated that the level of CXCL16/CXCR6 was significantly upregulated in the mouse PF model. Moreover, the level of p-STAT3 was also promoted. In addition, the mouse recombinant protein CXCL16 not only contributed to the proliferation of mouse LF cells but also induced the expression of p-STAT3 in LF cells. However, the effect of CXCL16 was deeply abolished by the STAT3 inhibitor AG490 in LF cells. Meanwhile, the antibody of CXCL16 deeply reduced the phosphorylation of STAT3 in lipopolysaccharide (LPS) cultured cells.

**Conclusions:**

All these results demonstrated that CXCL16 promoted the phosphorylation of STAT3 and further demonstrated that STAT3 was a critical component in CXCL16/CXCR6 signaling pathway. This research not only enhanced the comprehension of CXCL16 but also indicated its potential value as a target in the treatment for human PF.

## 1. Introduction

Pulmonary fibrosis (PF) belongs to the progressive lung disease, which is a respiratory disease with high morbidity and mortality [1, 2]. Numerous deaths are induced by PF every year all over the world. Due to the heterogeneity of PF, the outcome of the traditional approach for its treatment is far from satisfactory [1, 3]. Therefore, the novel therapy approach was urgently needed.

The transmembrane chemokine (C-X-C motif) ligand 16 (CXCL16) has played a key role in multiple biological processes. Previous report has demonstrated that CXCL16 involves in the pathogenesis of renal fibrosis and injury [4, 5]. Meanwhile, CXCL16 shows the potential value in the treatment for human papillary thyroid cancer [6]. Moreover, overexpression of CXCL16 has accelerated the proliferation and metastasis of lung cancer cells [7]. However, the biological function of CXCL16 is still not clear in PF.

It has been confirmed that C-X-C chemokine receptor 6 (CXCR6) is the receptor for CXCL16. The CXCL16/CXCR6 signaling pathway is involved in multiple biological activities. Previous report has indicated that CXCL16/CXCR6 signaling pathway associates with the development of lung cancer [8]. Moreover, activation of the CXCL16/CXCR6 axis leads to the progression of breast cancer and prostate cancer cells [9, 10]. However, the detailed function of CXCL16/CXCR6 signaling pathway in PF is still less identified.

It had been confirmed that the signal transducer and activator of transcription 3 (STAT3) is upregulated in lung fibroblasts and alveolar type II cells (ATII), which subsequently leads to lung fibrosis [11, 12]. Moreover, previous report has indicated that STAT3 contributes to profibrotic processes [13]. Therefore, inhibiting the activity of STAT3 shows the potential value in the treatment for PF.

In order to further examine the function of CXCL16 in the progression of PF, we constructed the PF model on mouse in the present research. Moreover, the mouse recombinant protein CXCL16 was used to examine the effect of CXCL16 on mouse lung fibroblasts L929 (LF) cells *in vitro*. In addition, a specific STAT3 inhibitor AG490 and lipopolysaccharide were used to determine the connection between CXCL16 and STAT3 in mouse LF cells. This research not only gained a deep comprehension of CXCL16 but also provided evidences to indicate its potential molecule pathway in mouse LF cells.

## 2. Materials and Methods

### 2.1. Blood

The blood sample of PF patients (*n* = 40) and normal people (*n* = 40) were obtained from Shanghai Sixth People's Hospital East Affiliated to Shanghai University of Medicine & Health Sciences. Then, the serum was isolated by centrifugation at 2000–3000 rpm for 20 min. Samples were subsequently snap-frozen by liquid nitrogen and stored at −80°C for further analysis. PF patients or non-PF volunteers were informed and written consent was obtained. This research was recognized by the independent ethics committee of Shanghai Sixth People's Hospital East Affiliated to Shanghai University of Medicine & Health Sciences.

### 2.2. PF Model

This section experiment was performed according to the institute's guidelines for animal experiments and was in agreement by the independent ethics committee of Shanghai Sixth People's Hospital East Affiliated to Shanghai University of Medicine & Health Sciences.

In this study, specific pathogen free (SPF) C57 male mice (20 g, *n* = 10; Slarc, Shanghai, China) were acclimatized in specific pathogen free conditions for one week. The PF model was induced by bleomycin according to the method of previous report [14]. In brief, mice were anesthetized by chloral hydrate (0.4 ml/100 g). Then, the model mouse (*n* = 5) was treated by bleomycin (5 mg/kg; Aladdin, Shanghai, China) through intratracheal instillation. The control mouse group (*n* = 5) was only treated by sterile saline. After being treated for four weeks, all mice were sacrificed by cervical dislocation under deep anesthesia. Blood was sampled from mouse eyes and used for examining the serum level of CXCL16 in peripheral bloods. The lung samples were fixed with formaldehyde and stored in liquid nitrogen for further analysis. All operations were performed to minimize suffering.

### 2.3. Cell Culture

This research was in agreement with the Declaration of Helsinki. The cell line used in this study was mouse L929, which was purchased from the cell bank of the Shanghai Biology Institute (Shanghai, China). Cells were cultured in DMEM medium (Trueline, USA) and grown in a 5% CO_2_ condition at 37°C. Then, cells were cultured by the mouse recombinant protein CXCL16 (Abcam, UK) and the STAT3 inhibitor AG490 (Selleck, USA) for 48 h with different concentrations, including 50, 100, and 200 ng/ml, respectively.

### 2.4. Histopathology Assay

In brief, all sample tissues were fixed in 10% formalin for 48 hours and subsequently embedded with paraffin. Then, the samples were cut into slice by using microtome (Leike, China). A series of Xylene baths and graded alcohols were utilized to deparaffinize and rehydrate. Then, hematoxylin and eosin (H&E) assay was performed for nuclear counterstaining after slices were reacted with diaminobenzidine (DAB) substrate. Three replicates were needed for each sample. Meanwhile, the Masson staining assay was performed according to the instruction of the manufacture (Leagene, Beijing, China). The myofiber appeared in red, whereas the light green or aniline blue represented collagenous fiber.

### 2.5. Cell Proliferation Assay

Cell proliferation profile was examined by using cell counting kit-8 (CCK-8) assay kits (SAB, USA). In Brief, cells were seeded in 96-well plates and cultured for 0, 24, 48, and 72 h; CCK-8 solution (1 : 10) was mixed to each well and incubated for 1 h. The OD value at wavelength 450 nm was measured by microplate reader (Pulangxin, China). Three replications were needed at each time point.

### 2.6. ELISA

In this section, we examined the serum level of CXCL16 in PF patients and mouse model. The serum samples were prepared as indicated above. Moreover, the protein level of TNF-*α*, IL-6, collagen I, and collagen III were quantified in different mouse L929 cells as mentioned. All ELISA kits used in this study were obtained from Bioscience (Shjgogo, China). The experimental procedures were performed according to the instructions of the manufacturer.

### 2.7. Biochemical Assay

The levels of hydroxyproline (HYP) were examined enzymatically with commercially available assay kits (njjcbio, China) according to the instructions of the manufacturer. The microplate reader (Pulangxin, China) was utilized to examine the absorbance of each well at 550 nm.

### 2.8. Real-Time PCR

Total RNA from different samples were isolated by using TRIzol Reagent (Invitrogen, USA). Then, cDNA synthesis kit (Fermentas, Canada) was used to reverse transcribe RNA into complementary DNA (cDNA). The program of the real-time PCR reaction was listed as follows: 95°C for 10 min followed by 40 cycles of 95°C for 15 s and 60°C for 45 s. GAPDH was used to normalized the gene expression. The relative gene expression was calculated using the 2^−*ΔΔCt*^ method. All data represented the mean of three replicates. Primer sequences are listed in Supplementary File 1.

### 2.9. Western Blot

RIPA lysis buffer (JRDUN, Shanghai, China) was used to extract protein as indicated. An enhanced BCA protein assay kit (Thermo Fisher, USA) was utilized to estimate the protein content. Total protein (25 *μ*g) was fractionated by using 10% SDS-PAGE and transferred to a nitrocellulose membrane (Millipore, USA) for 2 hours, which were probed at 4°C for 12 hours with the primary antibodies followed by incubation for 1 h at 37°C with the secondary antibody (antiserum-HRP tagged goat IgG anti-rabbit; 1 : 1000; Beyotime, China). An enhanced chemiluminescence system (Tanon, China) was utilized to quantify the content of protein expression. Each analysis was detected in triplicate. GAPDH was treated as the internal reference. Detailed information of the primary antibodies is provided in Supplementary Table 2.

### 2.10. Statistical Analysis

GraphPad Prism Version 7.0 (CA, USA) was used for statistical analyses. Data were presented by mean ± SD of at least three samples. ANOVA for multiple comparisons was used to determine statistical significance, which was accepted by *p* value < 0.05.

## 3. Results

### 3.1. Serum Level of CXCL16 Was Overexpressed in Human PF Patients

**Figure 1:**
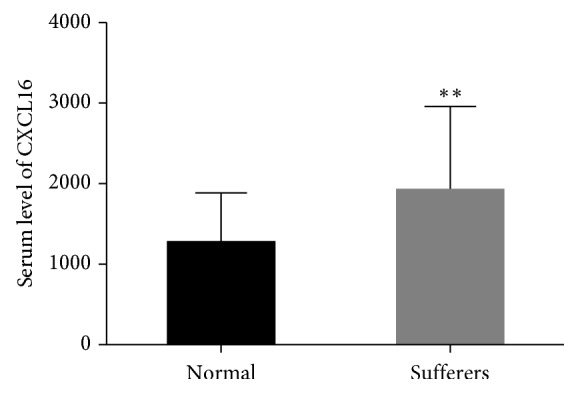
The serum level of CXCL16 was upregulated in PF sufferers (^*∗∗*^ *p* < 0.01 vs Normal).

The serum level of CXCL16 was examined by ELISA in 40 human PF patients and normal people. As shown in Figure 1, the serum level of CXCL16 was significantly increased in human PF patients compared with that of normal people.

### 3.2. PF Model Was Successfully Constructed on Mouse

**Figure 2:**
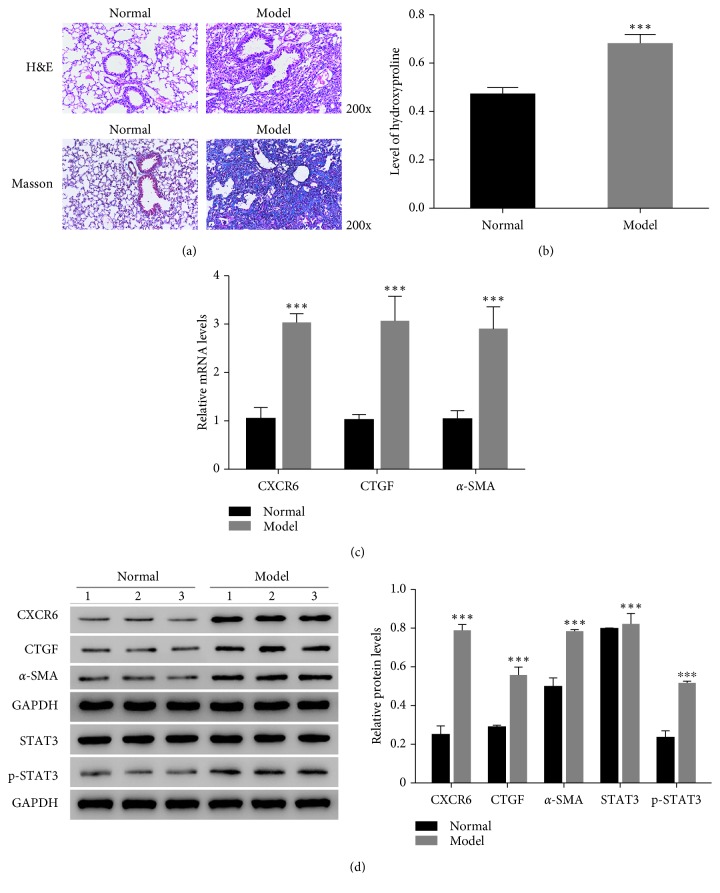
The PF model was successfully constructed in mice. (a) The lung tissues of normal and model mice were stained by hematoxylin and eosin (H&E) and Masson, respectively, 200x. (b) The production of HYP was increased in lung tissues of PF model mice (^*∗∗∗*^ *p* < 0.001 vs Normal). (c) The mRNA level of CXCR6, CTGF, and *α*-SMA was upregulated in lung tissues of PF model mice (^*∗∗∗*^ *p* < 0.001 vs Normal). (d) The protein level of CXCR6, CTGF, *α*-SMA, STAT3, and p-STAT3 was promoted in lung tissues of PF model mice (^*∗∗∗*^ *p* < 0.001 vs Normal).

In order to further examine the function of CXCL16 on PF, we constructed the mouse PF model by using bleomycin. As shown in Figure 2(a), the alveolar structure of normal mouse was visible, and the boundary was clear. However, the alveolar wall of model mice was more thickened than that of normal mouse. Meanwhile, alveolar structure of model mouse was deeply destroyed and the alveolar space become narrowed and fused. In addition, the collagenous fiber was rapidly accumulated in model mice. Hydroxyproline (HYP) is reported as a biomarker for idiopathic PF [15, 16]. In this part, we also examined the production of HYP in the PF model. Clearly, the production of HYP was significantly upregulated in the model group compared with that of the normal group (Figure 2(b)).

Connective tissue growth factor (CTGF) is induced by TGF-beta, which plays a key role in the pathogenesis of fibrosis [17]. Previous report has demonstrated that overexpression of CTGF induces the fibrosis of kidney [18]. Meanwhile, the level of *α* smooth muscle actin (*α*-SMA) is reported as a useful predictor in the development of fibrosis [19]. Thus, we also examined the mRNA and protein level of CXCR6, CTGF, and *α*-SMA in the mouse PF model. As shown in Figures 2(c) and 2(d), it was clear to identify that the levels of CXCR6, CTGF, and *α*-SMA were significantly upregulated in the model group. Moreover, we found the phosphorylation of STAT3 was also promoted in the mouse PF model. Taken together, these results demonstrated the PF model was successfully constructed in mice.

### 3.3. CXCL16 Was a Positive Factor in the Progression of PF

**Figure 3:**
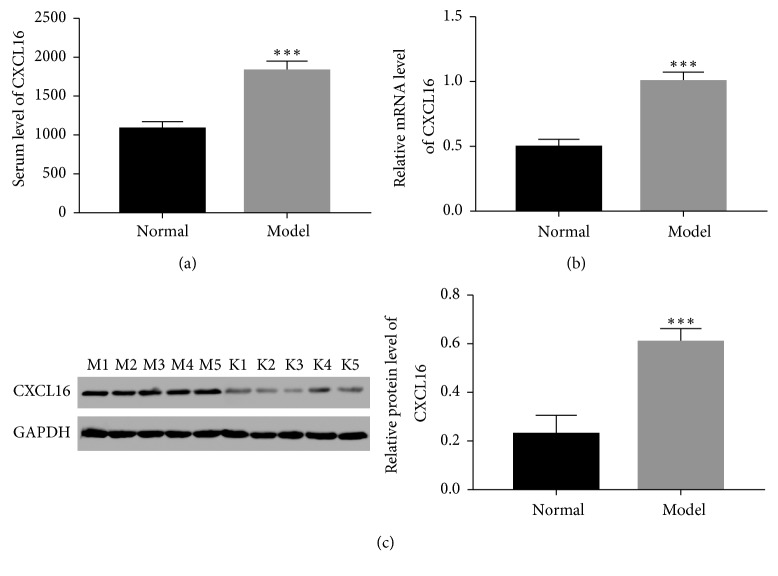
The level of CXCL16 was increased in mice PF model. (a) The serum level of CXCL16 was upregulated in the mouse PF model group (^*∗∗∗*^ *p* < 0.001 vs Normal). (b, c) mRNA and protein levels of CXCL16 were upregulated in lung tissues of PF model mice, respectively (^*∗∗∗*^ *p* < 0.001 vs Normal). M, the model mice that were treated by bleomycin; N, the normal mice treated by sterile saline.

Next, we examined the serum level of CXCL16 in the PF model. According to the ELISA results, the serum level of CXCL16 of model mice was much higher than that of normal mice (Figure 3(a)). Moreover, real-time PCR and western blot were used to qualify the mRNA and protein content of CXCL16. Both the mRNA and protein levels of CXCL16 were significantly overexpressed in the model group compared with that of the normal group (Figures 3(b) and 3(c)). Therefore, these results demonstrated that CXCL16 was a positive factor in the progression of PF.

### 3.4. Mouse Recombinant Protein of CXCL16 Promoted the Proliferation of Mouse LF Cells

**Figure 4:**
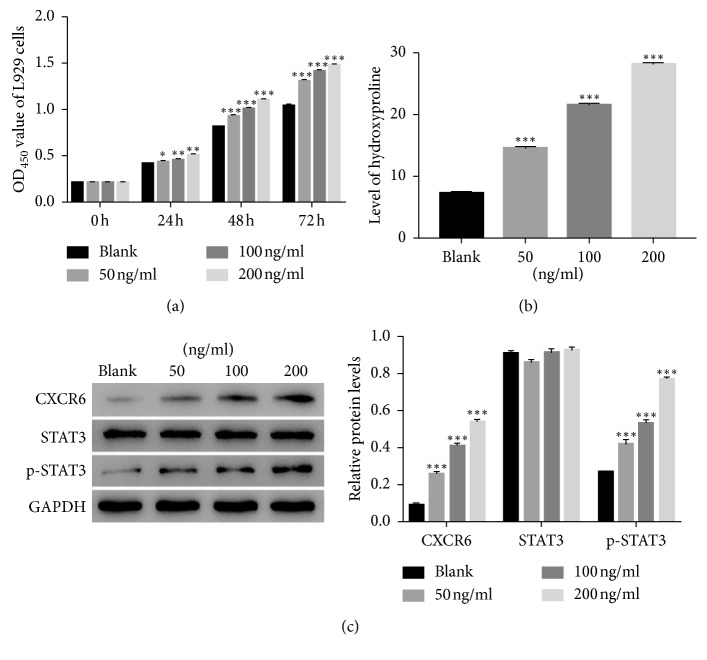
The mouse recombinant protein of CXCL16 contributed to the growth of mouse LF cells. (a) Cell proliferation was detected at 0, 24, 48, and 72 hours after being cultured by mouse recombinant protein CXCL16 with different concentration as indicated (^*∗*^ *p* < 0.05 vs Blank, ^*∗∗*^ *p* < 0.01 vs Blank, ^*∗∗∗*^ *p* < 0.001 vs Blank). (b) The production of HYP was promoted in mouse LF cells that were cultured by mouse recombinant protein of CXCL16 with different concentrations as indicated (^*∗∗∗*^ *p* < 0.001 vs Blank). (c) The protein level of CXCR6, STAT3, and p-STAT3 was increased by the protein of CXCL16 in mouse LF cells as indicated (^*∗∗∗*^ *p* < 0.001 vs Blank).

In order to further examine the function of CXCL16, the mouse recombinant protein CXCL16 was used to culture mouse LF cells with different concentrations, including 50, 100, and 200 ng/ml. The cell counting kit-8 (CCK-8) assay was used to analyze the proliferation rate of cells with different treatments. As the concentration of CXCL16 increased, the proliferation rate of mouse LF cells was gradually upregulated (Figure 4(a)). We also quantified the production of HYP in the recombinant protein of CXCL16 cultured cells. It was clearly identified that CXCL16 promoted the production of HYP in mouse LF cells (Figure 4(b)). Moreover, western blot was utilized to examine the protein content of CXCR6, STAT3, and p-STAT3 in different treated cells. The protein content of CXCR6 was significantly increased in the mouse recombinant protein CXCL16 cultured cells. Meanwhile, the phosphorylation of STAT3 was also remarkably activated by the recombinant protein of CXCL16 in mouse LF cells (Figure 4(c)).

### 3.5. Effect of CXCL16 Was Inhibited by a Specific STAT3 Inhibitor AG490 on Mouse LF Cells

**Figure 5:**
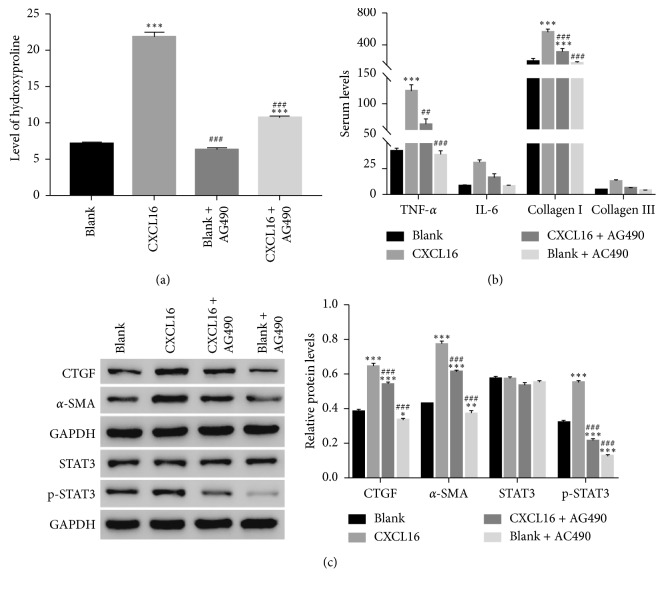
The effect of CXCL16 was inhibited by the STAT3 inhibitor AG490 on mouse LF cells. (a) The inhibitor AG490 suppressed the production of HYP in CXCL16 treated cells (^*∗∗∗*^ *p* < 0.001 vs Blank; ###*p* < 0.001 vs CXCL16). (b) The serum level of TNF-*α*, IL-6, collagen I, and collagen III detected in different cells as indicated. (c) The protein level of CTGF, *α*-SMA, STAT3, and p-STAT3 was inhibited by AG490 in CXCL16 cultured cells (^*∗*^ *p* < 0.05 vs Blank, ^*∗∗*^ *p* < 0.01 vs Blank, ^*∗∗∗*^ *p* < 0.001 vs Blank; ###*p* < 0.001 vs CXCL16).

To further determine the correlation between CXCL16 and STAT3 in mice LP cells, a specific STAT3 inhibitor AG490 was used to culture mouse LF cells. The production of HYP was significantly increased in recombinant CXCL16 cultured cells (Figure 5(a)). However, the level of HYP was deeply suppressed by the inhibitor AG490 in mouse LF cells.

It has been reported that inhibiting the expression of proinflammatory cytokine tumor necrosis factor-*α* (TNF-*α*) and IL-6 suppress the progression of PF [20, 21]. Moreover, the upregulation of collagen is also identified as a hallmark for PF [22]. Interestingly, the serum level of TNF-*α*, IL-6, and collagen I was also deeply inhibited by the STAT3 inhibitor AG490 in CXCL16 treated cells (Figure 5(b)). Meanwhile, the protein level of CTGF and *α*-SMA were also significantly increased in CXCL16 treated cells. However, both of them were deeply downregulated by the STAT3 inhibitor AG490. Meanwhile, the phosphorylation of STAT3 was also decreased in AG490 treated cells (Figure 5(c)). Taken together, all these results demonstrated that CXCL16 might target STAT3 in mouse LF cells.

### 3.6. Effect of Lipopolysaccharide Was Reduced by the Antibody of CXCL16 on Mouse LF Cells

Lipopolysaccharide (LPS) is reported as an endotoxin. The accumulation of LPS often leads to the acute lung injury [23, 24]. Therefore, the inhibitor of LPS is an attractive target in the treatment for acute lung injury. Moreover, LPS also stimulates the expression of CXCL16 in T cells [25]. In order to analyze the connection between LPS and CXCL16, in this study, the mouse LF cells were cultured by LPS (100 ng/ml) and then treated by the antibody of CXCL16 with different concentrations (50 and 100 ng/ml, respectively).

**Figure 6:**
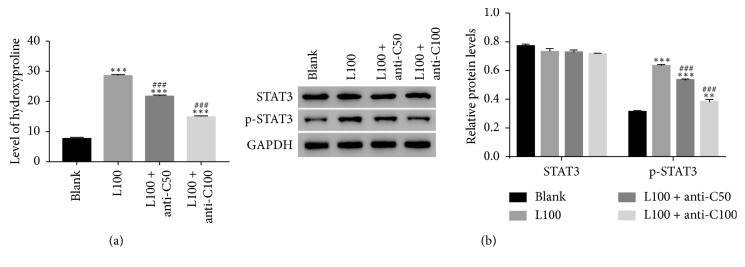
The antibody of CXCL16 reduced the effect of LPS on mouse LF cells. (a) The antibody of CXCL16 deeply suppressed the production of HYP in LPS cultured cells (^*∗∗∗*^ *p* < 0.001 vs Blank; ###*p* < 0.001 vs L100). (b) The phosphorylation of STAT3 was inhibited by the antibody of CXCL16 in LPS cultured cells (^*∗∗*^ *p* < 0.01 vs Blank, ^*∗∗∗*^ *p* < 0.001 vs Blank; ##*p* < 0.01 vs L100, ###*p* < 0.001 vs L100).

As presented in Figure 6(a), the production of HYP was remarkably upregulated in LPS cultured cells. Interestingly, the antibody of CXCL16 deeply suppressed the production of HYP in LPS treated cells. Moreover, the protein content of p-STAT3 was also significantly promoted by LPS in mouse LF cells. However, this effect of LPS was also deeply abolished by the antibody of CXCL16 (Figure 6(b)). Taken together, these results suggested that the antibody of CXCL16 abolished the effect of LPS on mouse LF cells.

## 4. Discussion

PF has reduced the supply of oxygen to the blood and subsequently led to the dysfunction of lung and respiratory failure [26]. Due to the character of heterogeneity, the outcome of the treatment for PF is commonly different. Therefore, the novel effective therapies are eagerly desired. In the present study, we constructed the PF model on mouse and analyzed the effect of the mouse recombinant protein CXCL16 on mouse LF cells. Our results indicated that CXCL16 had the potential value as a biomarker in the diagnosis for PF.

Previous report has demonstrated that CXCL16/CXCR6 signaling pathway promotes the progression of liver fibrosis [27]. Moreover, CXCL16/CXCR6 has contributed to the pathogenesis of renal fibrosis [28]. In the present study, the expression of CXCL16 and CXCR6 was upregulated in the mouse PF model and mouse LF cells. Therefore, these results not only indicated the positive feedback between CXCL16 and CXCR6 but also demonstrated the critical role of CXCL16/CXCR6 signaling pathway in the progression of PF. Moreover, the CXCL16/CXCR6 pathway might be a potential target in the prevention of organ fibrosis.

Previous report has illuminated that CTGF is overexpressed in liver fibrotic lesions [29]. Suppression of CTGF is reported as an effective therapy in the prevention and reversal of fibrosis [30]. Meanwhile, *α*-SMA is identified as an indicator in predicting fibrosis [31]. Moreover, it has been confirmed that inhibiting the activity of *α*-SMA attenuates lung fibrosis [32]. In this study, our results indicated that the expression of CTGF and *α*-SMA was also upregulated in the mouse PF model and promoted by the mouse recombinant protein CXCL16 in mouse LF cells. However, both of them were deeply suppressed by the STAT3 inhibitor AG490 in mouse LF cells. Taken together, all these results demonstrated that STAT3 might be the target for CXCL16 in mouse LF cells. Moreover, the STAT3 inhibitor AG490 was a potential promising agent in the treatment of PF.

It has been reported that the major function of LPS is achieved though regulating the p38MAPK-STAT3 axis signal [33]. Our results indicated that the effect of the mouse recombinant protein CXCL16 on mouse LF cells was significantly abolished by the STAT3 inhibitor AG490. More importantly, the antibody of CXCL16 also inhibited the phosphorylation of STAT3 in LPS cultured cells. Moreover, CXCL16 also mediates the activity of STAT3 in rheumatoid arthritis synovial fibroblasts [34]. In this study, our analyses also obtained the similar results. Therefore, our analysis further demonstrated that STAT3 might be a target for CXCL16 in mouse LF cells.

## 5. Conclusion

In this study, we constructed the mouse PF model and examined the effect of the mouse recombinant protein CXCL16 on mouse LF cells. This study not only enhanced the comprehension of CXCL16 but also indicated its potential signaling pathway in PF.

## Data Availability

The data used to support the findings of this study are included within the article.

## Supplementary Materials

Supplementary Materials Supplementary File 1: primer sequence information. Supplementary Table 2: the primary antibodies information.
