# Supplementary material for: CXCL16 Induces the Progression of Pulmonary Fibrosis through Promoting the Phosphorylation of STAT3
Source: Can Respir J. 2019 Jul 10;2019:2697376. doi: 10.1155/2019/2697376 (PMC6652085; doi:10.1155/2019/2697376)
Supplement: Supplementary Materials — Supplementary File 1: primer sequence information. Supplementary Table 2: the primary antibodies information. [file 2697376.f1.docx]

# Supplementary File1：Primer sequence information

# 1.1 Mus musculus chemokine (C-X-C motif) ligand 16, mRNA (cDNA clone MGC:28190 IMAGE:3988542), complete cds

BC019961.1

Primer F 5' ACCGCAGGGTACTTTGGATC 3'

Primer R 5' CTCAGGGGTCTGGGTACTGG 3'

Pos: 141-366

Amplified product: Size: 226 bps

**1.2 Mus musculus glyceraldehyde-3-phosphate dehydrogenase (Gapdh), transcript variant 1, mRNA**

NM_001289726.1

Primer A 5' CTGCCCAGAACATCATCC 3'

Primer B 5' CTCAGATGCCTGCTTCAC 3'

Pos: 691-887

Amplified product: Size: 197 bps

# 1.3 Rattus norvegicus C-X-C motif chemokine ligand 16 (Cxcl16), mRNA

NM_001017478.

Primer F 5' TATTTAGCCCAAGCCAACC 3'

Primer R 5' ACCCTGTCTCAAACAAACC 3'

Pos: 1305-1407

Amplified product: Size: 103 bps

# 1.4 Rattus norvegicus C-X-C motif chemokine receptor 6 (Cxcr6), mRNA

NM_001102587.2

Primer F 5' GGTTCTTCCTGCCATTGC 3'

Primer R 5' TGCTATCGCCTCTGTCAC 3'

Pos: 623- 858

Amplified product: Size: 236 bps

# 1.5 Rattus norvegicus cellular communication network factor 2 (Ccn2), mRNA

NM_022266.2

Primer F 5' TAGCCTCAAACTCCAAAC 3'

Primer R 5' ATAAACTGCCTCCCAAAC 3'

Pos: 1969- 2258

Amplified product: Size: 290 bps

**1.6 Rattus norvegicus actin, alpha 2, smooth muscle, aorta (Acta2), mRNA**

NM_031004.2

Primer F 5' AACACGGCATCATCACCAAC 3'

Primer R 5' CACAGCCTGAATAGCCACATAC 3'

Pos: 278- 480

Amplified product: Size: 203 bps

**1.7 Rattus norvegicus glyceraldehyde-3-phosphate dehydrogenase (Gapdh),mRNA**

NM_017008.4

Primer F    5' GGAGTCTACTGGCGTCTTCAC 3'

Primer R    5' ATGAGCCCTTCCACGATGC 3'

Pos:  357-593

Amplified product:   Size: 237 bps

| **Supplementary Table 2: The primary antibodies information** | | | | |
| --- | --- | --- | --- | --- |
| Antibody name | Molecular. Weight | Article Number | Source | Dilution Factor |
| CXCR6 | 40KDa | Ab191442 | Abcam, UK | 1:1000 |
| STAT3 | 88KDa | Ab119352 | Abcam, UK | 1:5000 |
| p-STAT3 | 88KDa | Ab76315 | Abcam, UK | 1:5000 |
| CTGF | 36KDa | Ab6992 | Abcam, UK | 1:1000 |
| α-SMA | 42KDa | Ab5694 | Abcam, UK | 1:1000 |
| CXCL16 | 28 kDa | ab84434 | Abcam, UK | 1:1000 |
| GAPDH | 37 KDa | #5174 | CST | 1:2000 |
